# Warmer temperatures advance flowering in a spring plant more strongly than emergence of two solitary spring bee species

**DOI:** 10.1371/journal.pone.0218824

**Published:** 2019-06-24

**Authors:** Sandra Kehrberger, Andrea Holzschuh

**Affiliations:** Department of Animal Ecology and Tropical Biology, Biocenter, University of Würzburg, Würzburg, Germany; Universite du Quebec a Chicoutimi, CANADA

## Abstract

Climate warming has the potential to disrupt plant-pollinator interactions or to increase competition of co-flowering plants for pollinators, due to species-specific phenological responses to temperature. However, studies focusing on the effect of temperature on solitary bee emergence and the flowering onset of their food plants under natural conditions are still rare. We studied the effect of temperature on the phenology of the two spring bees *Osmia cornuta* and *Osmia bicornis*, by placing bee cocoons on eleven grasslands differing in mean site temperature. On seven grasslands, we additionally studied the effect of temperature on the phenology of the red-list plant *Pulsatilla vulgaris*, which was the first flowering plant, and of co-flowering plants with later flowering. With a warming of 0.1°C, the abundance-weighted mean emergence of *O*. *cornuta* males advanced by 0.4 days. Females of both species did not shift their emergence. Warmer temperatures advanced the abundance-weighted mean flowering of *P*. *vulgaris* by 1.3 days per 0.1°C increase, but did not shift flowering onset of co-flowering plants. Competition for pollinators between *P*. *vulgaris* and co-flowering plants does not increase within the studied temperature range. We demonstrate that temperature advances plant flowering more strongly than bee emergence suggesting an increased risk of pollinator limitation for the first flowers of *P*. *vulgaris*.

## Introduction

Species-specific phenological shifts in response to climate warming can alter the temporal overlap among mutualistic but also antagonistic partners, and as a consequence also the structure of whole communities [1,2]. For animal pollinated angiosperms, which constitute 78% of all angiosperms in the temperate zones [3], wild- and honeybees are the main pollinators [4]. For both plants and pollinators a temporal mismatch with their interaction partners can have negative consequences for survival and reproductive output and can furthermore affect population dynamics [5,6]. Whereas for plants temporal mismatches with pollinators can lead to reduced visitation rates and reduced pollen deposition, for pollinators a temporal mismatch with their forage plants can reduce the availability of nectar and pollen [5]. Negative consequences of a temporal mismatch may be particularly high at the beginning of the season, when other potential interaction partners are not yet available that could replace the original interaction partner [7]. On the other hand, species may benefit from temporal mismatch, if their interaction with competitors is desynchronized by non-parallel phenology shifts. However, non-parallel phenology shifts of co-occurring plant species can also increase competition, e.g. if those shifts result in a prolonged period of flowering overlap with co-flowering plants and thus enhanced competition for pollinators [8,9]. Therefore the right timing of phenological events is important to maximize the temporal overlap with mutualists, but also to minimize the temporal overlap with competitors [10,11]. However, if interaction partners respond to different cues, different combinations of cues or the same cues but to different extents, a decoupling of temporal synchrony could arise [1,12].

Temperature is an important trigger of wild bee emergence [7,13] and often the main driver of flowering phenology in temperate regions [5,7]. However, the flowering phenology of plants can also be affected by other environmental cues, like precipitation, photoperiod or time of snowmelt [14,15]. For bees the temperature experienced during overwintering influences the timing of emergence, with spring bees incubated under warmer temperatures emerging earlier than spring bees incubated under colder temperatures [16–18]. Also many plant species advance flowering onset in response to climate warming [12,19], however, the degree of response varies greatly among species [20]. Studies investigating the effects of climate warming on plant-pollinator synchrony differ in their results, probably due to species-specific differences in the response to environmental cues. Some studies showed that bees have advanced their phenology more strongly than plants in response to climate change [19,21–23], others found that plants have advanced more strongly [7,24–26] or found no difference in the phenological shift of plants and bees [13,27]. However, most of these studies focused on the synchrony between plant phenology and activity of bumble bees [13,24–26] or used museum collections providing flight activity data to study the synchrony between plant phenology and flight activity of solitary bees [21,22,27], whereas field studies on the synchrony between plant phenology and solitary bee emergence are still scarce (but see [7]). A disadvantage of using flight activity data is that it can be biased by the detectability of flying bees, which depends on their abundance and the presence of mutualistic species [28]. So the absence of flowering plants at the beginning of the season or the low abundance of bees during the first days of emergence or during the last days of the flying season can lead to missed bees. Missed individuals would not only underestimate the actual flying season length of the studied bees but could also mask a potential temporal mismatch between the first bees and the first flowers if bees are active before flowering onset of the first flowers but are not detected due to missing floral resources. These problems can be avoided by recording emergence dates instead of flight activity. Field studies on the effect of temperature on the timing of bee emergence and flowering can help to understand how an environmental cue affects the synchronisation of pollinators and plants in a variable environment, and provide the basis for predicting effects of future climate warming on plant-pollinator interaction. Plant-pollinator interactions are not only affected by species-specific shifts of flowering phenology, but also by changes in the flowering duration [5]. The flowering duration of a plant species can be compressed or elongated if flowering onset and end shift non-uniformly [20]. Furthermore, species-specific shifts of flowering duration can alter the temporal co-flowering patterns in sequentially flowering plant species, which can also modify interspecific interactions [9,20,29].

Besides temporal mismatches among interacting species due to climate warming, temporal mismatches among mating partners of a species could also occur. Many pollinators show protandry, which is the emergence of males prior to females [30]. Protandry is supposed to maximize reproductive success for males and to reduce the risk of pre-reproductive death for females [31]. A laboratory study showed that in some solitary bee species warmer overwintering temperatures reduce the degree of protandry [18]. On the contrary, long-term phenological data from museum specimen records showed that warmer temperatures shift the flying season of solitary bees at a similar rate for both sexes, indicating no change in the degree of protandry [27]. However, experimental evidence on the effect of temperature on the degree of protandry in solitary bees under field conditions is still lacking.

In this study we tested the effect of temperature on the timing of emergence in the spring bee species *Osmia cornuta* and *Osmia bicornis*, by placing bee cocoons on eleven calcareous grasslands that differed in the mean temperature. We assessed if temperature alters the degree of emergence protandry of the studied bees. Furthermore, we tested the influence of temperature on the flowering onset, flowering end and flowering duration of seven populations of the red-list perennial spring plant *Pulsatilla vulgaris*, which is the first herbal plant species to flower on the studied grasslands and on the flowering onset of the co-flowering plant species of *P*. *vulgaris*. Specifically, we asked (1) how temperature shifts the timing of emergence and the degree of protandry in *O*. *cornuta* and *O*. *bicornis*, (2) how temperature shifts flowering onset and end of *P*. *vulgaris* and flowering onset of co-flowering plant species and (3) whether temperature affects the flowering duration of *P*. *vulgaris* and the time span of *P*. *vulgaris* flowering in the absence of co-flowering plant species. We hypothesize that warmer temperatures affect the phenology of male and female bees, *P*. *vulgaris* and co-flowering plants, to different extents.

## Material and methods

### Study sites

We studied eleven calcareous grasslands in an area of about 840 km^2^ in the vicinity of Würzburg (49° 48´ N, 9° 56´ E), Germany, with a minimal distance of 2.5 km between study sites (S1 Table). Grasslands were at least 1 ha in size and the only sites in the region where *Pulsatilla vulgaris* populations of more than 50 individuals were expected to occur. However, on one site we found only 15 *P*. *vulgaris* individuals and on three sites no flowering individuals and therefore used seven sites in the plant analyses and eleven sites in the bee analyses. The population sizes of *P*. *vulgaris* on the study sites ranged between 50 and 600 individuals.

Grasslands differed by their exposition to sun and had differing mean temperatures. We hourly recorded air temperature with two temperature loggers per site (iButton temperature logger DS1922L, Maxim Integrated, USA; resolution: 0.0625°C). Loggers were fixed on two posts, 90 cm above ground, underneath the bee tubes (see below) and facing to south. Temperature recordings started on 6^th^ February 2015 and ended on 30^th^ May 2015. For each site, we calculated the mean temperature of the two loggers and the whole recording period. On one site, one logger failed between 6^th^ February and 21^st^ March 2015 and on another site, one logger failed between 6^th^ February and 27^th^ March 2015. For the two sites and time periods we used only the temperature obtained from one logger. The difference in mean temperature was 1.05°C between the warmest and the coldest of the eleven sites and 0.28°C between the warmest and the coldest *P*. *vulgaris* site.

### Timing of bee emergence

We studied the two spring bee species *Osmia cornuta* and *Osmia bicornis* (Hymenoptera: Apiformes: Megachilidae). Both species overwinter as imagines in the cocoon, are univoltine and polylectic. *O*. *cornuta* males emerge from beginning of March to end of April, while females emerge from beginning of March to beginning of June. *O*. *bicornis* males emerge from beginning of April to mid-May, females from beginning of April to end of July [32]. The foraging range of *O*. *cornuta* is 100–200 m [33] and up to 600 m for *O*. *bicornis* [34]. During the flight period of *O*. *cornuta*, *Pulsatilla vulgaris* was the only food plant flowering on the study sites. At landscape scales, the only other potential food plant at this time was *Salix caprea*, which occasionally occurred within the bee foraging range. During the flight period of *O*. *bicornis*, several food plants started flowering.

Timing of bee emergence was studied by placing 1100 cocoons of *O*. *cornuta* and 1100 cocoons of *O*. *bicornis* on the study sites. Bee cocoons were purchased from WAB Mauerbienenzucht (Konstanz, Germany), which is a commercial supplier of wild bee species. Bee cocoons were stored in a climate chamber at constant 4°C between October 2014 and 19^th^ January 2015. Then, in the lab, bee cocoons were filled in plastic tubes (length: 25.5 cm, diameter: 7 cm), whose open ends were closed with gauze (mesh width: 1mm). Each tube contained 50 bee cocoons. In total there were 22 tubes with *O*. *cornuta* and 22 with *O*. *bicornis* cocoons. Filled tubes were stored at an exterior area at the University of Würzburg until the 4^th^ or 5^th^ February 2015, when the tubes were brought to the study sites.

To predict phenological events of insects, like emergence, degree-day models can be used [35]. Those models take into account the length of a period (e.g. in days) in which a certain temperature threshold has been exceeded and the temperature experienced during that period. After a certain value in degree-days has been reached the phenological event takes place [35]. Previous studies on emergence dates of several solitary bee species have suggested that bees only accumulate degree-days above a temperature-threshold between 8°C– 14°C, and after a specific starting date of degree-day accumulation [7,17,36]. Accumulation of degree-days does not start before the starting date even if temperatures are above the temperature-threshold before the starting date [7]. Pre-wintering temperatures have not been found to affect the timing of emergence in solitary bees [16,37].

We placed two tubes per species in each site. Tubes were fixed on two wooden posts at one meter above ground with open ends directed east-west. One tube per species was fixed on the north side of a post and the other one on the south side of the other post. The two posts were 5 to 100 m apart from each other, depending on the size of the study site. Tubes were checked for emerged bees between 6^th^ February and 4^th^ March 2015 every fourth to tenth day on each site and between 4^th^ March and 15^th^ May 2015 every second to third day on each site. On 29^th^ May, all tubes were checked for the last time, however, no more bees had emerged. Remaining cocoons were then removed. For each emerged bee, we recorded species, sex and date of recording, which was taken as the date of emergence. For the analyses, we used for each site, species and sex the first and the last Julian date of emergence as well as the abundance-weighted mean date of emergence, which is the arithmetic mean of all days on which a bee of this species and sex had emerged on this site, weighted by its abundance on each date on this site [28]. The degree of protandry is the difference between females and males of a species in the first date, the last date and the abundance-weighted mean date of emergence. Due to a severe storm, we lost one tube containing *O*. *bicornis* cocoons on one site, after only one male bee had emerged. Thus for this site we used only recordings from one tube for analysis of the last Julian date of emergence for males and of the first, abundance-weighted mean and last Julian date of emergence for females of *O*. *bicornis*, as well as for the analysis of the degree of protandry for abundance-weighted mean and last date of emergence.

### Plant phenology

*Pulsatilla vulgaris* (Ranunculaceae) was the first and only plant species flowering on the study sites when the study bees started to emerge. *P*. *vulgaris* is a perennial herb restricted to calcareous grasslands and listed as a threatened plant species on the red lists of threatened plant species of Germany and Bavaria [38,39]. Reproduction is vegetative as well as sexual with bees being the main flower visitors [40]. The most abundant wild flower visitors were *O*. *cornuta* and *O*. *bicornis*, responsible for 39% of the visits by wild bees, followed by bumblebees (37%) and bees of the genus *Andrena* (24%). The managed honey bee *Apis mellifera* was responsible for 53% of the total bee visits.

Flowering phenology of *P*. *vulgaris* populations and of co-flowering plant species was recorded between 6^th^ February and 4^th^ March 2015 every fourth to tenth day on each site and every second to third day on each site from 4^th^ March to 8^th^ May 2015. We stopped recording after detecting no more *P*. *vulgaris* flowers on all sites at consecutive recording dates. For each recording we walked across the study sites to look for *P*. *vulgaris* flowers and then defined a variable transect of 100 m^2^ containing the highest abundance of *P*. *vulgaris* flowers. For each transect the abundance of *P*. *vulgaris* flowers was estimated. For the analyses, we used for each site the first and the last Julian date of *P*. *vulgaris* flowering as well as the abundance-weighted mean date of flowering, which is the arithmetic mean of all dates on which *P*. *vulgaris* flowered at this site, weighted by its abundance on each date on this site [28]. The flowering duration of each *P*. *vulgaris* population was calculated as the difference between the Julian date of flowering end and the Julian date of flowering onset of *P*. *vulgaris* on the site.

We also recorded the Julian date when the first plant species other than *P*. *vulgaris* started to flower. During the flowering period of *P*. *vulgaris* we recorded three up to ten co-flowering plant species per study site, however plant species identity differed partly between sites. In total, we recorded 20 different co-flowering plant species. We hypothesize that the co-flowering plants compete with *P*. *vulgaris* for pollinators and therefore calculated the time span of *P*. *vulgaris* flowering in the absence of co-flowering plant species, which represents the flowering period in which only *P*. *vulgaris* flowered, as the difference between the first co-flowering plant species and *P*. *vulgaris* in their Julian date of flowering onset.

### Statistical analyses

To test how temperature affects emergence dates and protandry of *O*. *cornuta* and *O*. *bicornis*, we used linear models with number of emerged bee individuals and site temperature as predictors and phenological variables or degree of protandry as response variables. Phenological variables were the first, the abundance-weighted mean and the last Julian date of emergence. The number of emerged bee individuals only had a significant positive effect on the abundance-weighted mean emergence of *O*. *bicornis* females, in all other models there was no significant effect and hence we excluded the number of emerged bee individuals from those models. Emergence models were calculated separately for each sex and species, protandry models for each species.

To test how temperature affects the flowering phenology and the total flowering duration of *P*. *vulgaris* we used linear models with population size and site temperature as predictors. However, there was no effect of population size and therefore we excluded population size from the models. We also tested if temperature has an effect on the flowering onset of co-flowering plant species and the time span of *P*. *vulgaris* flowering in the absence of co-flowering plant species with linear models with site temperature as predictor. We visually inspected model residuals for violation of assumptions of normality and homoscedasticity. All statistical analyses were performed using the software R [41].

## Results

Bees emerged from 83.0% of all *Osmia cornuta* cocoons and from 82.2% of all *Osmia bicornis* cocoons, with 44.8% males in *O*. *cornuta* and 57.3% males in *O*. *bicornis*. Emergence of *O*. *cornuta* started—depending on site—between 6^th^ and 18^th^ March for males and between 8^th^ and 20^th^ March for females, and ended between 25^th^ March and 7^th^ April for males and between 7^th^ and 18^th^ April for females. Emergence of *O*. *bicornis* started between 26^th^ March and 10^th^ April for males and between 10^th^ and 16^th^ April for females, and ended between 20^th^ April and 5^th^ May for males and between 4^th^ and 15^th^ May for females. In total, only 20 *O*. *cornuta* males and one *O*. *cornuta* female emerged before the flowering onset of *P*. *vulgaris* (all males on the four warmest *P*. *vulgaris* sites, the female on the third warmest site). Flowering of *Pulsatilla vulgaris* started between 13^th^ and 18^th^ March and ended between 18^th^ April and 05^th^ May. On the two warmest sites *P*. *vulgaris* started flowering before the first female bees had emerged. On all sites, the first male bees had been emerged before or emerged on the same day when *P*. *vulgaris* started flowering. Whereas warmer temperatures did not change the time lag between the first emerged *O*. *cornuta* male and the first *P*. *vulgaris* flower, the time lag between the last emerged female *O*. *bicornis* and the last *P*. *vulgaris* flower increased by 6.6 days per 0.1°C temperature increase (Table 1). The first co-flowering plant species, which all attracted bees and potentially competed with *P*. *vulgaris* for pollinators, were *Potentilla neumanniana* (three sites), *Viola* sp. (three sites) and *Primula veris* (one site) with a flowering onset between 9^th^ and 11^th^ April, and *Adonis vernalis* (one site) with a flowering onset on 3^rd^ April.

**Fig 1:**
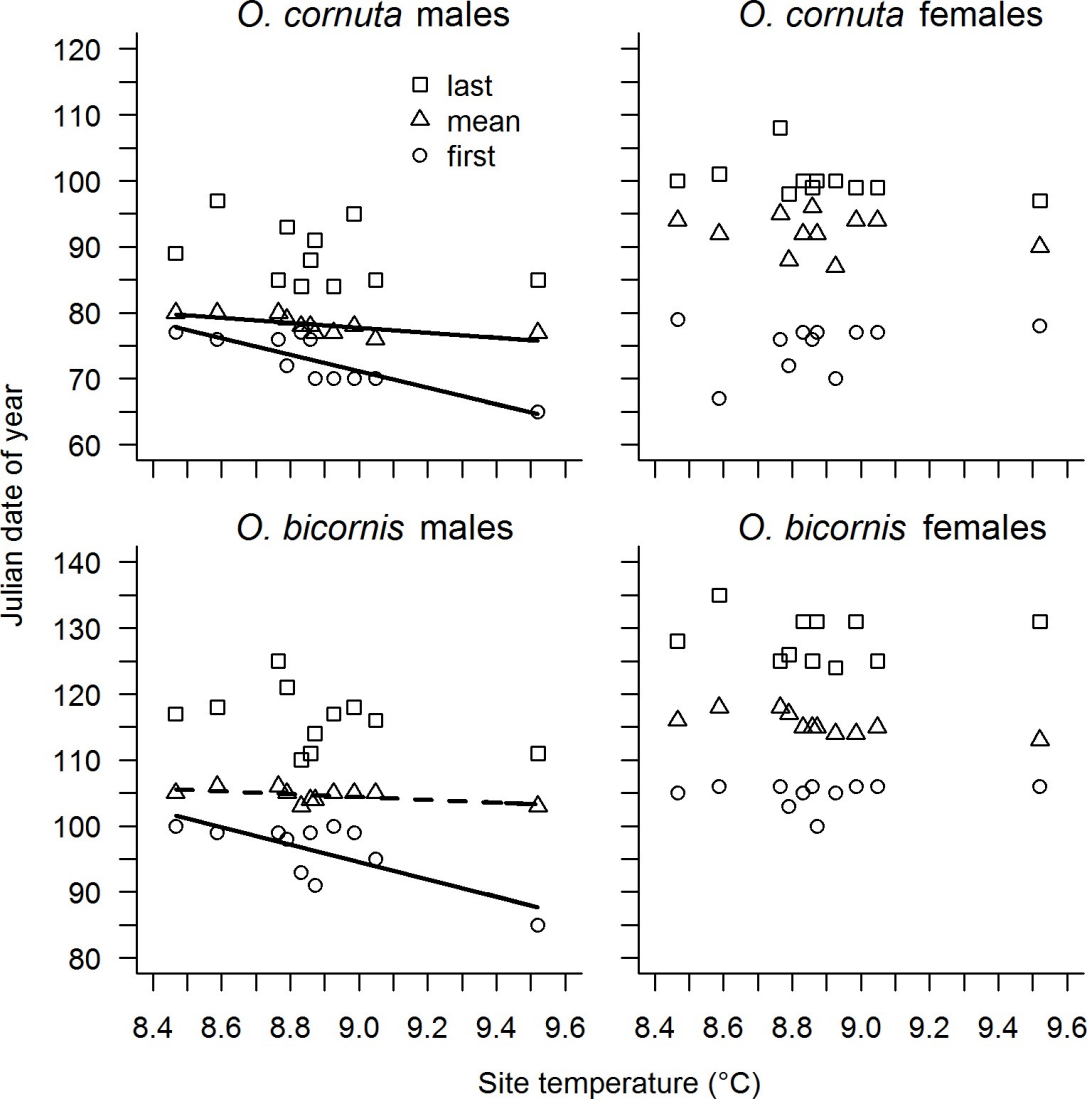
Site temperature effects on *O*. *cornuta* and *O*. *bicornis* emergence. Effect of site temperature on Julian date of first emergence (first), abundance-weighted mean emergence (mean) and last emergence (last) for *O*. *cornuta* males, *O*. *cornuta* females, *O*. *bicornis* males and *O*. *bicornis* females. Solid lines indicate significant relationships (*P* < 0.05), dashed lines marginal significant relationships (*P* < 0.1).

**Table 1:** Site temperature effects on *O*. *cornuta* and *O*. *bicornis* emergence on flowering phenology of *P*. *vulgaris* and co-flowering plants and on time lag between first bee and first flower and last bee and last flower. Slopes and 95% confidence levels (CL) are shown for models with p < 0.1.

Response	*df*	*F*	*P*	*slope*	*Lower CL (95%)*	*Upper CL (95%)*
*O*. *cornuta* males						
first emergence	9	22.6	**0.001**	- 1.2	- 1.8	- 0.7
wmd of emergence	9	9.8	**0.012**	- 0.4	- 0.6	- 0.1
last emergence	9	1.5	0.246	- 0.7	- 1.9	0.5
*O*. *cornuta* females						
first emergence	9	0.8	0.387	0.4	- 0.6	1.4
wmd of emergence	9	0.6	0.477	- 0.3	- 1.0	0.5
last emergence	9	1.9	0.203	- 0.4	- 1.2	0.3
*O*. *bicornis* males						
first emergence	9	11.3	**0.008**	- 1.3	- 2.2	- 0.4
wmd of emergence	9	4.2	0.070	- 0.2	- 0.5	- 0.0
last emergence	9	1.8	0.213	- 0.7	- 1.8	0.5
*O*. *bicornis* females						
first emergence	9	0.2	0.683	0.1	- 0.4	0.6
wmd of emergence *	8	0.5	0.503	- 0.4	- 0.8	- 0.1
last emergence	9	0.0	0.984	- 0.0	- 1.0	1.0
protandry *O*. *cornuta* (days)						
first emergence	9	13.1	**0.006**	1.6	0.6	2.7
wmd of emergence	9	0.1	0.742	0.1	- 0.7	0.9
last emergence	9	0.1	0.762	0.2	- 1.4	1.8
protandry *O*. *bicornis* (days)						
first emergence	9	17.8	**0.002**	1.4	0.7	2.2
wmd of emergence	9	3.4	0.097	- 0.2	- 0.5	0.0
last emergence	9	0.1	0.789	- 0.2	- 1.9	1.5
*P*. *vulgaris*						
flowering onset	5	14.2	**0.013**	- 1.9	- 3.3	- 0.6
wmd of flowering	5	8.9	**0.031**	- 1.3	- 2.4	- 0.2
flowering end	5	16.8	**0.009**	- 6.7	- 10.9	- 2.5
flowering duration	5	13.4	**0.015**	- 4.8	- 8.1	- 1.4
time span of *P*. *vulgaris* flowering in the absence of co-flowering plant species	5	0.9	0.390	1.5	- 2.7	5.7
co-flowering plant species						
flowering onset	5	0.1	0.763	- 0.4	- 3.7	2.9
time lag						
time lag between first *O*. *cornuta* male and first *P*. *vulgaris* flower	5	0.4	0.557	- 0.8	- 4.0	2.4
time lag between last *O*. *bicornis* female and last *P*. *vulgaris* flower	5	6.9	**0.047**	6.6	0.1	13.0

Effects of site temperature on the Julian date of first, abundance-weighted mean (wmd) and last emergence of *O*. *cornuta* and *O*. *bicornis* males and females, the degree of protandry, calculated as the difference between males and females in date of first, wmd and last emergence, flowering onset, wmd of flowering, flowering end, flowering duration and the time span of *P*. *vulgaris* flowering in the absence of co-flowering plant species and flowering onset of co-flowering plant species. The number of emerged bee individuals was significant in one model

(*) and was otherwise removed from the models.

A temperature increase of 0.1°C advanced the first emergence date of *O*. *cornuta* males by 1.2 days and the abundance-weighted mean date by 0.4 days, while for *O*. *bicornis* males, a temperature increase of 0.1°C advanced the first emergence date by 1.3 days but had no significant effect on the abundance-weighted mean date of emergence. Temperature did neither affect the first emergence date nor the abundance-weighted mean date of females in both species. The last emergence date was not affected by temperature in either sex or species (Table 1, Fig 1).

**Fig 2:**
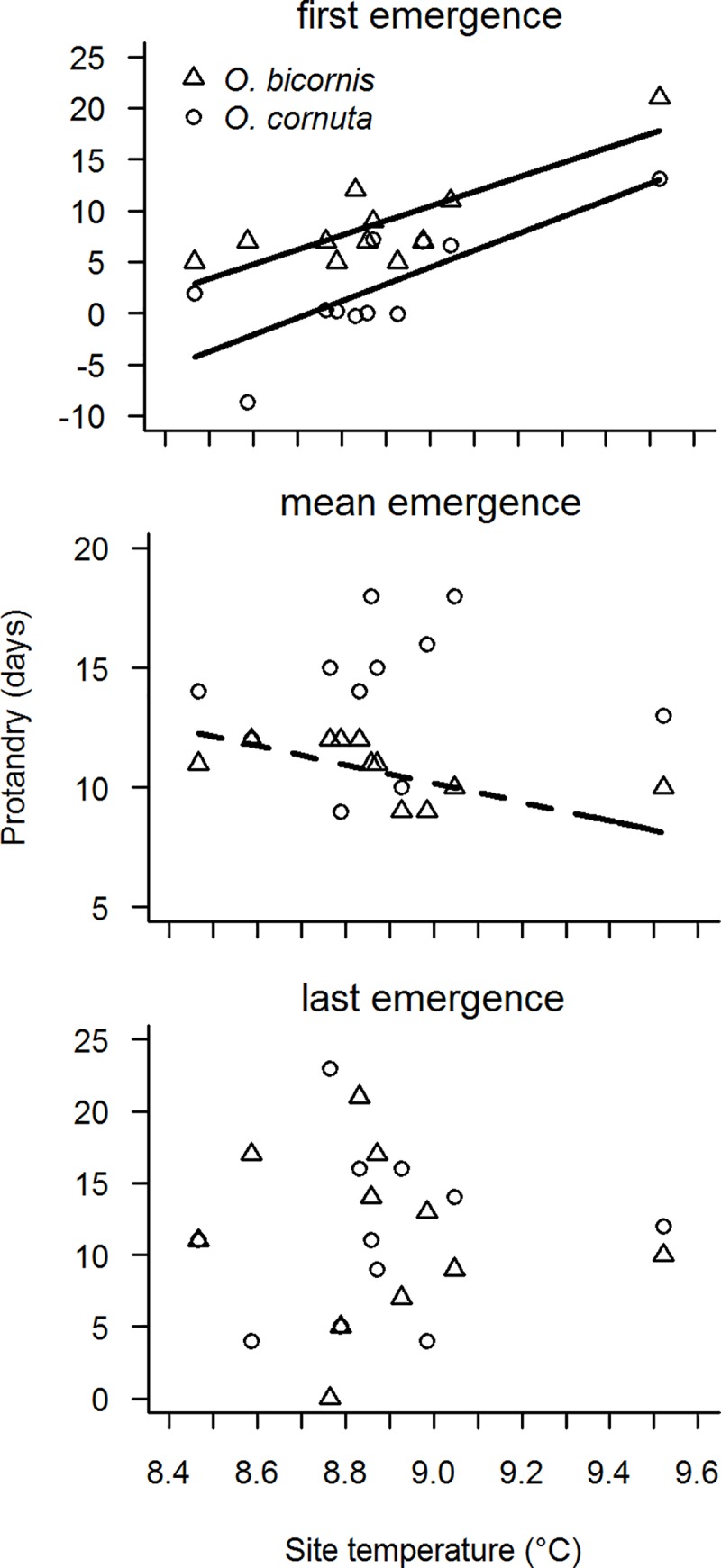
Site temperature effects on protandry levels of *O*. *cornuta* and *O*. *bicornis*. Effect of site temperature on the level of protandry calculated as the difference between females and males of *O*. *cornuta* and *O*. *bicornis* in first emergence,abundance-weighted mean emergence and last emergence. Solid lines indicate significant relationships (*P* < 0.05), dashed lines marginal significant relationships (*P* < 0.1).

The difference between the first dates of female and male emergence increased by 1.6 days per 0.1°C temperature increase for *O*. *cornuta* and by 1.4 days for *O*. *bicornis*. Differences in the abundance-weighted mean dates and in the last emergence dates between female and male of both species were not affected by temperature (Table 1, Fig 2).

**Fig 3:**
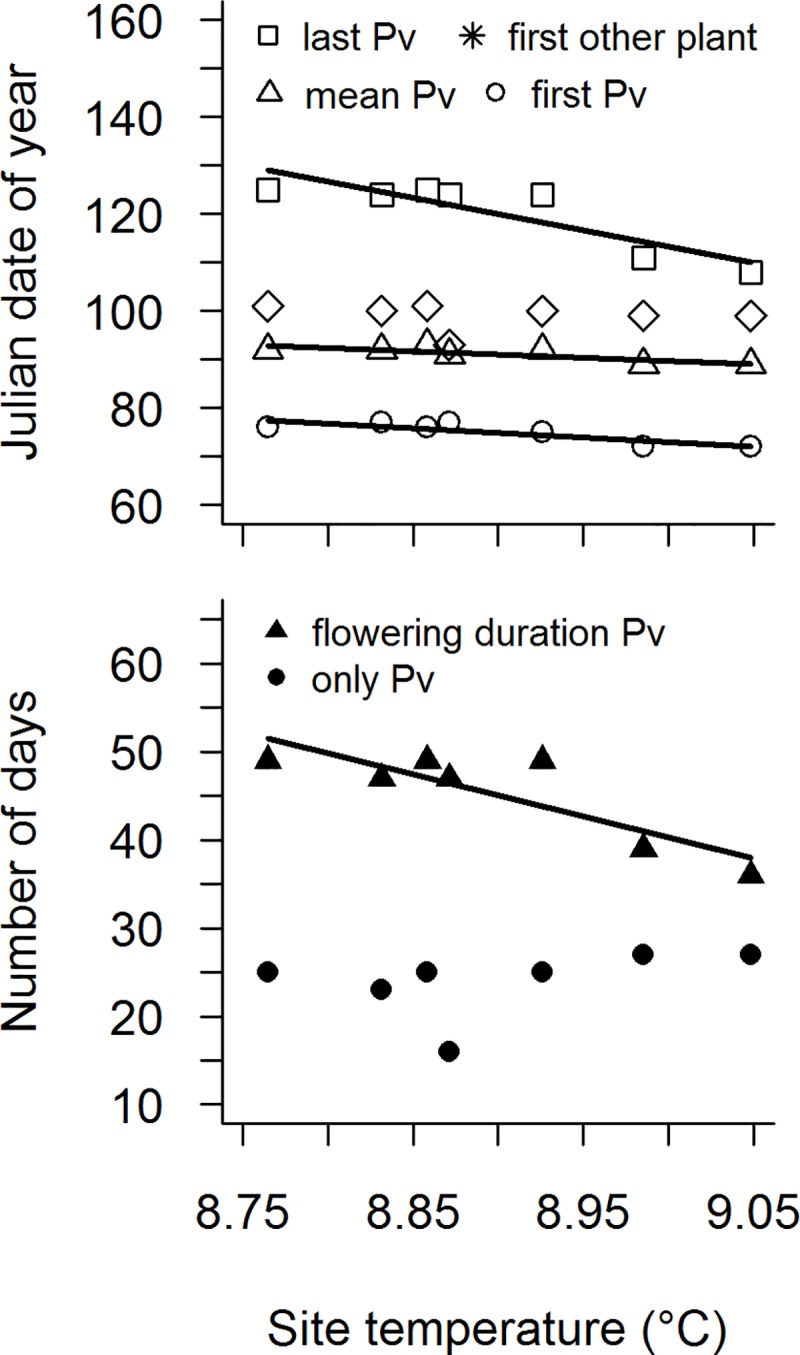
Site temperature effects on flowering phenology of *P*. *vulgaris* and co-flowering plants. Effect of site temperature on the Julian date of flowering onset (first Pv), abundance-weighted mean flowering (mean Pv) and flowering end (last Pv) for *P*. *vulgaris* and flowering onset of co-flowering plants (first other plant) and on the number of days of the time span of *P*. *vulgaris* flowering in the absence of co-flowering plant species (only Pv) and of the flowering duration of *P*. *vulgaris* (flowering duration Pv). Solid lines indicate significant relationships (*P* < 0.05).

Warmer temperatures advanced the flowering onset of *P*. *vulgaris* by 1.9 days per 0.1°C temperature increase, abundance-weighted mean flowering by 1.3 days and flowering end by 6.7 days. Temperature had no significant effect on the flowering onset of the first co-flowering plant species. A temperature increase of 0.1°C shortened flowering duration of *P*. *vulgaris* by 4.8 days, but did not alter the time span of *P*. *vulgaris* in the absence of co-flowering plant species (Table 1, Fig 3).

## Discussion

We showed that warmer temperatures accelerated the timing of emergence in the first *Osmia cornuta* and *Osmia bicornis* males, but not in the last. Female bees of both species were not affected by warmer temperatures. Flowering end of *Pulsatilla vulgaris* advanced 3.5 times stronger than flowering onset with warmer temperatures. Plant flowering shifted more strongly than bee emergence.

An increase of 0.1°C advanced the abundance-weighted mean emergence of *O*. *cornuta* males by 0.4 days. A less strong advance of mean emergence by about 0.1 days per 0.1°C increase was found in a laboratory study for *O*. *cornuta*, which might be explained by the study design with constant instead of naturally fluctuating temperatures as in our study [18]. Analyses of museum specimen records showed for the flight activity of North-American spring bees a similar shift towards earlier dates as we found, ranging between 0.18 to 0.50 days per 0.1°C increase [27]. However, for *Andrena nigroaenea* a much stronger advance of mean flight activity was found, with 0.74 days per 0.1°C increase for flight records, and 1.15 days per 0.1°C increase for museum collection data [22]. The differences in temperature dependence could be either species specific or due to the different methods used, with emergence data monitoring every individual of a population compared to flight activity data, which depends on the detectability of flying bees and can therefore lead to missed bees. The shift of the abundance-weighted mean flowering of *P*. *vulgaris*, which advanced by 1.3 days per 0.1°C increase, was three times as strong as the shift in abundance-weighted mean emergence of *O*. *cornuta* males.

The first date of emergence of *O*. *cornuta* and *O*. *bicornis* males as well as the first flowering date of *P*. *vulgaris* advanced more strongly than the abundance-weighted mean date of emergence and flowering with warmer temperatures, respectively. At the beginning of the season the first individuals in a population have the highest risk of a temporal mismatch with mutualistic interaction partners. In the study year, on the four warmest sites, 20 *O*. *cornuta* males and one female emerged before the first *P*. *vulgaris* flowered on the respective site. 97.8% of *O*. *cornuta* males and 99.9% of females emerged at or shortly after the flowering onset of *P*. *vulgaris* suggesting that bees and plants are currently well synchronized. We expect that the bee emergence dates we recorded did not differ from emergence dates of bees naturally nesting on the studied sites, because a reciprocal transplant experiment on trap-nesting bees showed that site of origin and therefore adaptations to site conditions had no effect on emergence phenology [7]. The advance of the first flowering date of *P*. *vulgaris*, with 1.9 days per 0.1°C increase was stronger than the advance of the first date of emergence of *O*. *cornuta* and *O*. *bicornis* males, with 1.2 and 1.3 days per 0.1°C increase, respectively. Our data thus suggest, that warm temperatures involve the risk that *P*. *vulgaris* starts flowering before the emergence of its main pollinator *O*. *cornuta*, which is the first cavity-nesting species on the studied sites. On the two warmest sites *P*. *vulgaris* already started flowering before the first *O*. *cornuta* female had been emerged. Female bees play a more important role in plant pollination than male bees which do not collect pollen for nest provision [42]. Pollinator limitation can result in reduced reproductive success and consequently have a negative effect on population size [43]. Plants ideally compensate for a temporal mismatch with their pollinators, by elongating their floral longevity, however, warm temperatures can restrict the ability to enhance floral longevity independently of pollination [44].

Also in our study we found that warmer temperatures shortened the flowering duration of *P*. *vulgaris* populations, due to a less strong advance of flowering onset compared to flowering end. We suggest that the shorter flowering duration of *P*. *vulgaris* populations with warmer temperatures is due to shorter floral longevities of individual flowers, induced by warmer temperatures. Contrary to our results, in early flowering montane plant species, warmer temperatures delayed the last date of flowering and lengthened the flowering season [20,29]. A compressed flowering period in response to warmer temperatures may negatively affect the reproductive success of a plant species, because it decreases the probability that the plant is visited by pollinators. Another way for *P*. *vulgaris* to compensate for pollinator limitation is to switch to vegetative reproduction if pollination fails [45], but this can reduce the genetic variability of the population and the adaptive plasticity to respond to environmental variation [46]. Besides compensation mechanisms implemented by the pollinator-limited plant itself, the plant can also mitigate negative effects resulting from non-parallel phenology shifts of plants and pollinators by shifting to other pollinators, which fulfil the same function [47]. Other early pollinators we could observe on *P*. *vulgaris* at the beginning of flowering were honeybees and bumble bee queens. Both, emergence of bumble bee queens from hibernation [27] and the first appearance of honeybees [19] have previously been found to advance with warmer temperatures, however, less strong than flowering onset of *P*. *vulgaris* in our study.

The last date of emergence in both bee species and sexes did not shift with warmer temperatures, whereas the last date of flowering of *P*. *vulgaris* advanced by 6.7 days per 0.1°C increase. We suggest that for *O*. *bicornis*, which emerges towards the end of the flowering period of *P*. *vulgaris*, the earlier flowering end of *P*. *vulgaris* with warmer temperatures, reduces the abundance of *P*. *vulgaris* flowers to forage on, with probable negative effects on the reproductive output of the bee if other food resources are also scarce.

Our results show not only a difference in the response to warmer temperatures between plants and pollinators, but also different responses between the sexes of bee species. In contrast to male bees of both species, female bees did not advance the first emergence in response to warmer temperatures within the studied temperature range. We suggest that this difference is due to different reproductive pressures and strategies of male and female bees. Whereas female bees are mostly monandrous, males are polygynous [48]. As a consequence males can only increase their reproductive success by mating with as many receptive, unmated females as possible, whereas the reproductive success of females depends on successful construction and provisioning of brood cells [48]. This imposes the selective pressure on male bees to emerge earlier than competing conspecific males and to increase the probability to encounter receptive, unmated females. We suggest that with warmer temperatures males that emerge at the beginning of the emergence period can increase their reproductive success by emerging even earlier. This leads to males in a population that encounter a risky reproductive strategy, despite the threats of bad weather conditions, absent flowering plants and desynchronization with females that come along with a disproportionate early emergence. However, with warmer temperatures this reproductive strategy can become even more risky if it coincides with a desynchronization with food plants, as a survival without food is more difficult at warmer temperatures than at colder temperatures [6]. The risk of desynchronization with females for the first emerged males is shown in the increased protandry of the first emerged individuals in both species, while protandry was not significantly affected by temperature when we focus on abundance-weighted mean emergence dates of the populations.

Whereas warmer temperatures advanced the flowering onset of *P*. *vulgaris*, flowering onset of its co-flowering plants did not change. However, this did not result in a significantly longer time span in which *P*. *vulgaris* flowered without co-flowering plant species. The reproductive success of a plant species can be strongly decreased by co-flowering plant species, which withdraw pollinators [49]. Thus, there may be a strong selective pressure on *P*. *vulgaris*, to start flowering prior to co-flowering plant species [10]. Our results indicate that—within the study range—warmer temperatures will not increase competition for pollinators between *P*. *vulgaris* and its co-flowering plants.

## Conclusion

As warm temperatures advance the emergence of spring bees less strongly than the flowering of an early plant, with warming temperatures early pollinator-dependent plants are at the risk to face pollinator limitation, with negative consequences for their reproductive success. On our four warmest study sites 20 *O*. *cornuta* males and one female emerged before the flowering onset of *P*. *vulgaris*, which could negatively affect the reproductive success of the bee population. However, for the threatened *P*. *vulgaris* with climate warming and its stronger shift in response to warmer temperatures compared to *O*. *cornuta*, this temporal mismatch could be reversed to the first flowers of *P*. *vulgaris* flowering without pollinators being present. Although phenological asynchrony of plants and pollinators can be mitigated by different compensation mechanisms, like alternative reproductive strategies, species complementarity or range shifts, warming temperatures impose a critical threat on mutualistic interaction partners. Non-parallel phenology shifts of bees and plant species can reduce the diversity and alter the composition of flowering plant communities, where bees can forage on during their flight season [21], with negative effects for bee larval development and reproductive success [6,50]. For the threatened plant species *P*. *vulgaris* a decrease of seed production, imposed by pollinator limitation, could reduce the genetic variability of the population through increased vegetative reproduction [46]. Reduced viability and reproductive success can negatively affect the population size and on a long-term scale even push a species to extinction.

## Supporting information

S1 Table Coordinates of study sites. (PDF)
