# Supplementary material for: Warmer temperatures advance flowering in a spring plant more strongly than emergence of two solitary spring bee species
Source: PLoS One. 2019 Jun 24;14(6):e0218824. doi: 10.1371/journal.pone.0218824 (PMC6590824; doi:10.1371/journal.pone.0218824)
Supplement: S1 Table — (PDF) [file pone.0218824.s001.pdf]

study site	latitude	longitude
1	50°00'19.5"N	9°48'21.5"E
2	49°49'44.6"N	9°51'07.0"E
3	49°51'55.9"N	9°47'20.8"E
4	49°46'52.0"N	9°48'27.3"E
5	49°46'42.0"N	9°41'59.4"E
6	49°58'27.9"N	9°49'13.9"E
7	49°51'15.8"N	9°50'03.3"E
8	49°58'11.8"N	9°47'12.1"E
9	49°59'54.9"N	9°42'06.7"E
10	50°01'38.1"N	9°47'58.0"E
11	49°47'26.2"N	9°45'14.9"E
